# Inclusion of nutritional counseling and mental health services in HIV/AIDS management: A paradigm shift

**DOI:** 10.1097/MD.0000000000035673

**Published:** 2023-10-13

**Authors:** Esther U. Alum, Emmanuel Ifeanyi Obeagu, Okechukwu P.C. Ugwu, Awotunde O. Samson, Adeyinka O. Adepoju, Mariam O. Amusa

**Affiliations:** a Department of Publications and Extension, Kampala International University, Kampala, Uganda; b Department of Biochemistry, Faculty of Science, Ebonyi State University, Ebonyi State, Nigeria; c Department of Medical Laboratory Science, Kampala International University, Kampala, Uganda; d Department of Biochemistry, Habib Medical School, IUIU, Kampala, Uganda; e Research Institute for Innovations, AME University, Monrovia, Liberia; f Department of Botany and Plant Biotechnology, University of Johannesburg, Johannesburg, South Africa.

**Keywords:** depression, HIV/AIDS, nutritional counseling, opportunistic infections, undernutrition

## Abstract

Human immunodeficiency virus (HIV) infection is a public health challenge that can degenerate into acquired immunodeficiency syndrome (AIDS) if not properly managed. HIV infection shortens life expectancy to about 5 to 10 years compared to noninfected individuals. People living with HIV/AIDS (PLWHA) are prone to several health challenges as a result of a deranged immune system culminating in high morbidity and mortality. Depression is a common feature of PLWHA. Depression heightens the emergence of opportunistic infections in HIV-infected individuals, accelerates the progression to AIDS, and increased suicidal tendencies, morbidity, and mortality. Food insecurity with its resultant undernutrition contributes to HIV/AIDS-related deaths. Undernourished PLWHA are more prone to opportunistic infections due to poor immunity. Interestingly, proper diet intake can boost immunity, slow the progression of AIDS and opportunistic infections, enhance body weight, and retard depression tendencies. Undernutrition can also be ameliorated by incorporating nutritional counseling and oral nutrient supplementation in routine HIV/AIDS checkups. Therefore, to increase HIV/AIDS management outcomes, the integration of nutrition counseling, dietary supplements, and mental health services should be embraced. Thus, HIV/AIDS care centers should amplify these services. In this article, we isolated relevant studies from various databases, illuminated the interwoven relationship between HIV/AIDS, depression, and undernutrition, and also reemphasized the need for adequate nutritional intervention in the battle against HIV/AIDS. Thus, this study provides a reawakening call to focus on incorporating nutritional guides and mental health care in HIV/AIDS management protocols.

## 1. Introduction

Human immunodeficiency virus (HIV) infection is a public health challenge with a bifold pervasiveness in Sub-Saharan Africa. Poorly managed HIV infection could degenerate into acquired immunodeficiency syndrome (AIDS) with its resultant immune compromise resulting in several diseases commonly called opportunistic infections.^[1,2]^ There has been a massive fight against HIV/AIDS through mass literacy campaigns championed by community health personnel, community, and religious leaders, and the provision of free antiretroviral drugs to severely affected regions. The decline in HIV/AIDS pandemic is attributed to these concerted efforts.^[3]^ People living with HIV/AIDS (PLWHA) are prone to several health challenges as a result of a deranged immune system culminating in high morbidity and mortality with cardiovascular diseases being the predominant cause of HIV-related deaths.^[4]^ Depression is a common attribute of PLWHA. Notably in 2017, 39% of HIV-positive individuals had depression with its accompanying poor health outcomes.^[5]^ Depression is a mental health challenge characterized by mood swings, low self-esteem, lethargy, suicidal thoughts, self-isolation, poor appetite, and sleeping difficulty. Regrettably, suicide is common in depressed patients.^[6]^ Food insecurity means grossly inadequate availability of food needed for healthy living.^[7]^ It is a global challenge with greater prevalence in developing countries.^[8,9]^ Food insecurity is tantamount to malnutrition. PLWHA are more prone to food insecurity with its resultant health challenges.^[10]^ More so, food insecurity is affiliated with an escalated likelihood of depression for PLWHA.^[11]^ Kinyanda et al^[12]^ evaluated the rate of depression in HIV-positive individuals who were food insecure in Uganda and reported a 2 to 3 fold higher chance of depression for HIV-positive individuals compared to their HIV-negative counterparts. This link between food insecurity and depressive disorders and adverse health outcome is also significant in developed regions. According to a study by Palar and his colleagues in the USA, PLWHA who were food insecure had 4-fold higher chances of presenting with depression unlike their food-secure counterparts.^[11]^ In this article, we did an extensive search of various electronic databases from 2009 to 2023 and isolated relevant studies. From these studies, we illuminated the interwoven relationship between HIV/AIDS, depression, food insecurity, and poor health outcome. We also spotlighted the saliency of adequate nutritional intake in improving health outcomes in PLWHA. Thus, this study provides a reawakening call to focus on incorporating nutritional guides before initiation and during antiretroviral therapy.

## 2. Depression and HIV/AIDS infection

Depression heightens the emergence of opportunistic infections in HIV-infected individuals and speedy advancement to AIDS. This could be attributed to an increase in viral loads and a decline in cluster of differentiation (CD4) counts.^[13]^ In a recent study to assess the prevalence of depression and associated factors among HIV/AIDS patients in Ethiopia, Seid and colleagues^[14]^ reported that PLWHA with low CD4 counts had a higher risk of depression than their counterparts with higher CD4 counts.^[14]^ This result corroborates previous reports in Malawi,^[15]^ Cameroon,^[16]^ and Uganda.^[17]^ More so, symptomatic HIV-positive patients are more prone to depression than asymptomatic patients.^[18]^ Also, non-disclosed HIV status increases the risk of depression.^[14]^ Further, depressed HIV-positive individuals have diminished adherence to antiretroviral drug intake leading to reduced therapeutic efficacy of antiretroviral drugs culminating in increased morbidity and mortality.^[19]^ Antiretroviral drugs play significant roles in the management of HIV/AIDS and the enhancement of health outcomes of PLWHA. Thus, nonadherence to antiretroviral intake escalates the severity of comorbidities affiliated with HIV/AIDS, leading to weight loss, more stigma, poor social life and consequently upturning depressive tendencies.^[20]^ In light of these facts, timely identification and treatment of depression in PLWHA can improve health outcomes in these individuals. Incorporating mental health services in HIV/AIDS management protocols is advocated.

Arsenious et al^[18]^ in their study to examine the relationship between HIV infection and depression reported that depression has a positive impact on HIV-infected individuals, accelerates progression to AIDS, and heightens the mortality rate. A related study by Nanni et al,^[21]^ reported that HIV infection enhances the emergence of depression and the importance of incorporating psychosocial interventions in HIV management. The role of dysregulated immunologic response in the perpetuation of depression during HIV infection cannot be ruled out. HIV infection can stimulate pro-inflammatory mediators like cytokines resulting in psychiatric symptoms.^[22]^ Increasing serotonin levels, a key mechanism of action of some antidepressant medications is hampered by HIV through the inhibition of tryptophan, the amino acid needed for the synthesis of serotonin. The inhibition of tryptophan leads to a decline in the effectiveness of serotonin escalating depression.

## 3. HIV/AIDS infection and undernutrition

The use of antiretroviral therapy (ART) has played a remarkable impact in the fight against HIV/AIDS. The majority of PLWHA have access to ART.^[23]^ Despite this remarkable feat, HIV infection shortens life expectancy to about 5 to 10 years compared to noninfected individuals.^[24]^ Malnutrition is basically grouped into undernutrition (low nutrients) and overnutrition (excess nutrients). Undernutrition is the commonest form especially in low- and middle-income countries. Undernutrition has been fingered as one of the key contributors to HIV/AIDS-related deaths and also promotes the emergence of other diseases called opportunistic infections in PLWHA.^[25]^ Undernutrition and HIV infection have a knitted relationship and promote each other.^[26]^ Undernutrition studies conducted in Tanzania and Ethiopia among PLWHA reported high levels of nutritional insufficiencies (27.7% and 26%, respectively) in PLWHA.^[27,28]^ Recently, Seid et al^[29]^ in their meta-analysis review of the prevalence of undernutrition and associated factors among HIV-infected adults taking ART in sub-Saharan Africa reported 23.74%. These figures are relatively high and of serious health concern, given that undernutrition escalates the likelihood of developing opportunistic infections and increased mortality and morbidity.^[25]^ According to Seid et al,^[29]^ reduced CD4^+^ counts, male gender, advanced disease stage, younger age, delayed initiation of ART, and nonadherence to ART escalate the risk of undernutrition in PLWHA.

One of the key features of undernutrition is anemia with its associated health challenges leading to increased incidence of frequent hospitalization.^[30–32]^ Loss of appetite, increased energy need, and poor nutrient absorption are factors that exacerbate undernutrition in PLWHA.^[33]^ These factors promote loss of weight and skinny appearance which can lead to self-stigmatization, social isolation, and depression. Accordingly, proper diet intake can boost immunity, slow the progression of AIDS and opportunistic infections, enhance body weight, and retard depression tendencies. Food insecurity has been identified as one of the causes of undernutrition especially in PLWHA.^[27,28]^ Sub-Saharan Africa is one of the regions bedeviled by food insecurity and HIV infection.^[34,35]^ Thus the need to intensify nutritional support programs in this region.

Undernutrition also reduces the efficacy of ART leading to reduced chances of survival in PLWHA.^[36]^ Poor feeding habits by HIV-infected individuals could predispose them to undernutrition. Consumption of low-calorie foods, and inadequate intake of protein-rich foods, fruits, and vegetables can make PLWHA have insufficient nutrients exacerbating undernutrition and its associated consequences.^[37]^ Food insecurity, poverty, diarrhea, and vomiting can aid poor feeding habits.^[38]^ Rawat and colleagues^[37]^ identified poor diet quality as a contributor to low CD4 counts, anemia, and a predictor of death among PLWHA in Uganda. Food insecurity has also been reported among PLWHA on the shore of Lake Victoria, Kenya.^[39]^

## 4. Tackling food insecurity and depression in PLWHA: Nutritional intervention

Food insecurity can diminish HIV viral load suppression in HIV-infected individuals thereby enhancing the emergence of depression.^[40]^ Further, the reduced CD4 count during HIV infection in food insecure patients could heighten depressive disorder risks in PLWHA.^[41]^ Weight loss, a common feature of food insecure PLWHA, presents its own undesirable health outcomes.^[42]^ The role of optimum body weight in the maintenance of a healthy life is well documented.^[43–45]^ Severe underweight and its resultant skinny appearance escalate the risk of social isolation, stigma, and depression.^[46]^ Malnourished PLWHA are more prone to opportunistic infections due to poor immunity. Loss of appetite and diarrhea hampers nutrient availability in PLWHA. Thus, the importance of early diagnosis, treatment of these duos, and adherence to antiretroviral medications could palliate nutrient deficiency in PLWHA. Adequate consumption of a balanced diet contributes to healthy living and PLWHA is no exemption.^[47]^ Food nutrients especially those from plant origin are good sources of vitamins, minerals, and other components required for healthy living.^[48–50]^ Hong and colleagues reported that protein-energy-fortified nutrient supplementation at antiretroviral initiation upregulates nutritional status and immunity in PLWHA in their review of 15 studies conducted from 2000 to 2015 across sub-Saharan Africa.^[51]^

However, proper monitoring of protein-energy supplementation is necessary to avert the dangers of excessive calorie intake. Dysregulated protein-energy metabolism can predispose to hyperglycemia and abnormal lipid profile.^[52,53]^ The hallmark of excessive blood sugar levels is hyperglycemia and oxidative stress thus, increasing the risk of other diseases like cardiovascular diseases,^[45,54]^ liver diseases,^[55,56]^ kidney malfunction,^[57]^ and inflammatory disorders.^[58,59]^ Cardiovascular diseases have been identified as the topmost cause of death in PLWHA.^[4]^ Interestingly, fruits and vegetables have been proven to help in the management of hyperglycemia,^[45]^ cardiovascular diseases,^[60]^ inflammatory disorders,^[61]^ and other diseases.^[62]^ Thus, adequate intake of fruits, vegetables, and other plant-based supplements could promote the survival rate of PLWHA. Nutritional counseling has also proven effective in boosting the health outcomes of PLWHA.^[63]^ Notably, Jesson and Leroy reported that dietary counseling with oral nutritional supplements enhances health outcomes in PLWHA.^[64]^

## 5. Conclusion

PLWHA are at increased risk of undernutrition which can be ameliorated by incorporating nutritional counseling and oral nutrient supplementation in their routine HIV/AIDS checkups. Depression is also a common feature in PLWHA. Depressed HIV-infected individuals are more prone to undernutrition. Therefore, to increase HIV/AIDS management outcomes, the integration of nutritional counseling, dietary supplements, and mental health services should be embraced. Thus, HIV/AIDS care centers should amplify these services.

## Author contributions

**Conceptualization:** Emmanuel Ifeanyi Obeagu, Esther A. Alum.

**Methodology:** Emmanuel Ifeanyi Obeagu, Esther A. Alum, Okechukwu P.C. Ugwu.

**Resources:** Emmanuel Ifeanyi Obeagu, Esther A. Alum, Okechukwu P.C. Ugwu.

**Supervision:** Emmanuel Ifeanyi Obeagu.

**Validation:** Emmanuel Ifeanyi Obeagu.

**Visualization:** Emmanuel Ifeanyi Obeagu, Esther A. Alum.

**Writing – original draft:** Emmanuel Ifeanyi Obeagu, Esther A. Alum, Okechukwu P.C. Ugwu, Awotunde O. Samson, Adeyinka O. Adepoju, Mariam O. Amusa.

**Writing – review & editing:** Emmanuel Ifeanyi Obeagu, Esther A. Alum, Okechukwu P.C. Ugwu, Awotunde O. Samson, Adeyinka O. Adepoju, Mariam O. Amusa.
